# Neutrophil swarming and extracellular trap formation play a significant role in Alum adjuvant activity

**DOI:** 10.1038/s41541-016-0001-5

**Published:** 2017-01-23

**Authors:** J. Stephen, H. E. Scales, R. A. Benson, D. Erben, P. Garside, J. M. Brewer

**Affiliations:** Institute of Infection, Immunity and Inflammation College of Medical, Veterinary and Life Sciences Sir Graeme Davies Building, University of Glasgow, 120 University Place, Glasgow, G12 8TA Scotland UK

## Abstract

There are over 6 billion vaccine doses administered each year, most containing aluminium-based adjuvants, yet we still do not have a complete understanding of their mechanisms of action. Recent evidence has identified host DNA and downstream sensing as playing a significant role in aluminium adjuvant (aluminium hydroxide) activity. However, the cellular source of this DNA, how it is sensed by the immune system and the consequences of this for vaccination remains unclear. Here we show that the very early injection site reaction is characterised by inflammatory chemokine production and neutrophil recruitment. Intravital imaging demonstrates that the Alum injection site is a focus of neutrophil swarms and extracellular DNA strands. These strands were confirmed as neutrophil extracellular traps due to their sensitivity to DNAse and absence in mice deficient in peptidylarginine deiminase 4. Further studies in PAD4−/− mice confirmed a significant role for neutrophil extracellular trap formation in the adjuvant activity of Alum. By revealing neutrophils recruited to the site of Alum injection as a source of the DNA that is detected by the immune system this study provides the missing link between Alum injection and the activation of DNA sensors that enhance adjuvant activity, elucidating a key mechanism of action for this important vaccine component.

## Boosting immunity: How alum NETS improved vaccination

Researchers have identified the mechanism of action in which aluminium hydroxide (alum) boosts the efficacy of co-administered vaccines. In this study, James Brewer and colleagues from the University of Glasgow, UK, studied the effects of alum co-administration in a mouse model, finding that the compound induces immune cells, called neutrophils, to swarm around the site of immunization and produce neutrophil extracellular traps (NETs). NETs, webs of DNA and cell contents formed under certain conditions of neutrophil cell death, immobilize pathogens and promote their elimination. This study builds on previous research and demonstrates that alum artificially stimulates this pathway to boost the adaptive immune response to vaccine antigens, increasing their immunogenicity. The authors also suggest further study into neutrophil components as potential therapeutic agents.

## Introduction

Following its discovery in 1926,^1^ aluminium hydroxide (Alum) has been unique in its prolonged use as an adjuvant in human vaccines. Alum is known to induce a Th_2_ immune response, characterised by the production of interleukin (IL)-4 and murine IgG_1_ antibodies.^2^ However, despite several theories being proposed, the mechanism of action of Alum remains unclear. Glenny originally suggested that Alum functioned through the formation of a depot at the site of immunisation, resulting in the slow release of antigen and/or sustained tissue inflammation.^3^ However, our recent work clearly demonstrates that removal of the Alum injection site, as soon as 2 h after immunisation, had no impact on the resulting adaptive immune response.^4^ While the ability of Alum to trigger innate immune responses via the NLRP3 inflammasome and the subsequent production of proinflammatory cytokines has been highlighted,^5–7^ the role of the inflammasome in mediating Alum function is controversial, with others showing that key components of the inflammasome, such as Nlrp3 and caspase 1, are dispensable for adjuvant activity.^8,9^ More recently, interest has grown in the role of endogenous danger signals, such as host DNA, in Alum adjuvant function. It has been demonstrated that host DNA is accessible to enzyme (DNase) degradation following Alum immunisation and plays a role in driving antigen-specific T-cell response and B-cell response via DNA sensors such as IRF3.^10^ Similarly, sensing of host DNA by STING1 was shown to drive enhanced antigen presentation via Dendritic Cells ﻿(DCs) and prolonged T cell–DC interactions following Alum immunisation.^11^ Overall, these studies suggest that release of host DNA plays a pivotal role in the adjuvant function of Alum. However, the cellular source of this host DNA and the mechanism of DNA release remain unclear. Here we analyse the very early responses to Alum at the injection site and demonstrate that neutrophils are the principal cell recruited within 2 h of injection. Intravital imaging revealed neutrophil swarming and cell death focussed around Alum, and the presence of DNA strands within the tissue. These strands were subsequently confirmed as neutrophil extracellular traps (NETs) via their sensitivity to DNase treatment. NETs were also absent in PAD4-deficient mice, which also displayed markedly reduced immune responses following Alum injection. These studies demonstrate that the mechanism of neutrophil death plays an important role in the adjuvant activity of Alum, and explain how the cellular reaction at the injection site drives the liberation of host DNA, which subsequently impacts on adjuvant activity.

## Results

### Alum rapidly establishes an inflammatory milieu following injection

**Fig. 1:**
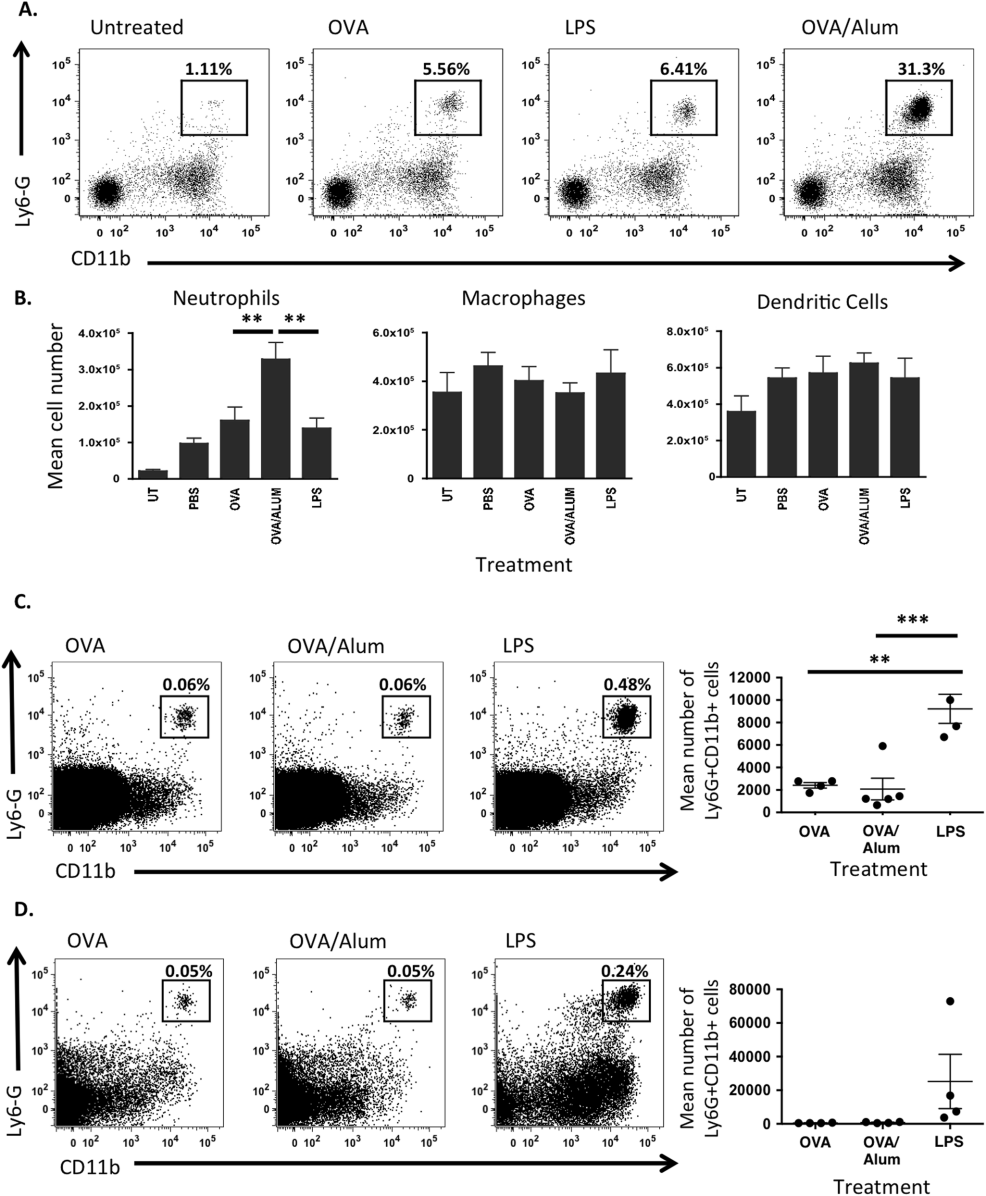
Alum induces neutrophil recruitment at the site of immunisation but not at the dLN within 2 h. **a** Representative flow cytometry plots depicting the recruitment of Ly6G^+^CD11b^+^ neutrophils to the site of alum immunisation. **b** Mean number (±SEM) of Ly6G^+^ neutrophils, F4/80^+^ macrophages and CD11c^+^ dendritic cells recruited within 2 h to the alum injection site (UT; Untreated, PBS; PBS-injected). Groups contained six animals and data are representative of three independent experiments. **c** Representative flow cytometry plots and *bar charts* depicting the mean number of Ly6G^+^CD11b^+^ neutrophils (±SEM) recruited to the draining cervical LN 2 h and **d** 24 h post immunisation. Groups contained three to five animals and data are representative of three independent experiments. **P < 0.01, ***P < 0.001

**Fig. 2:**
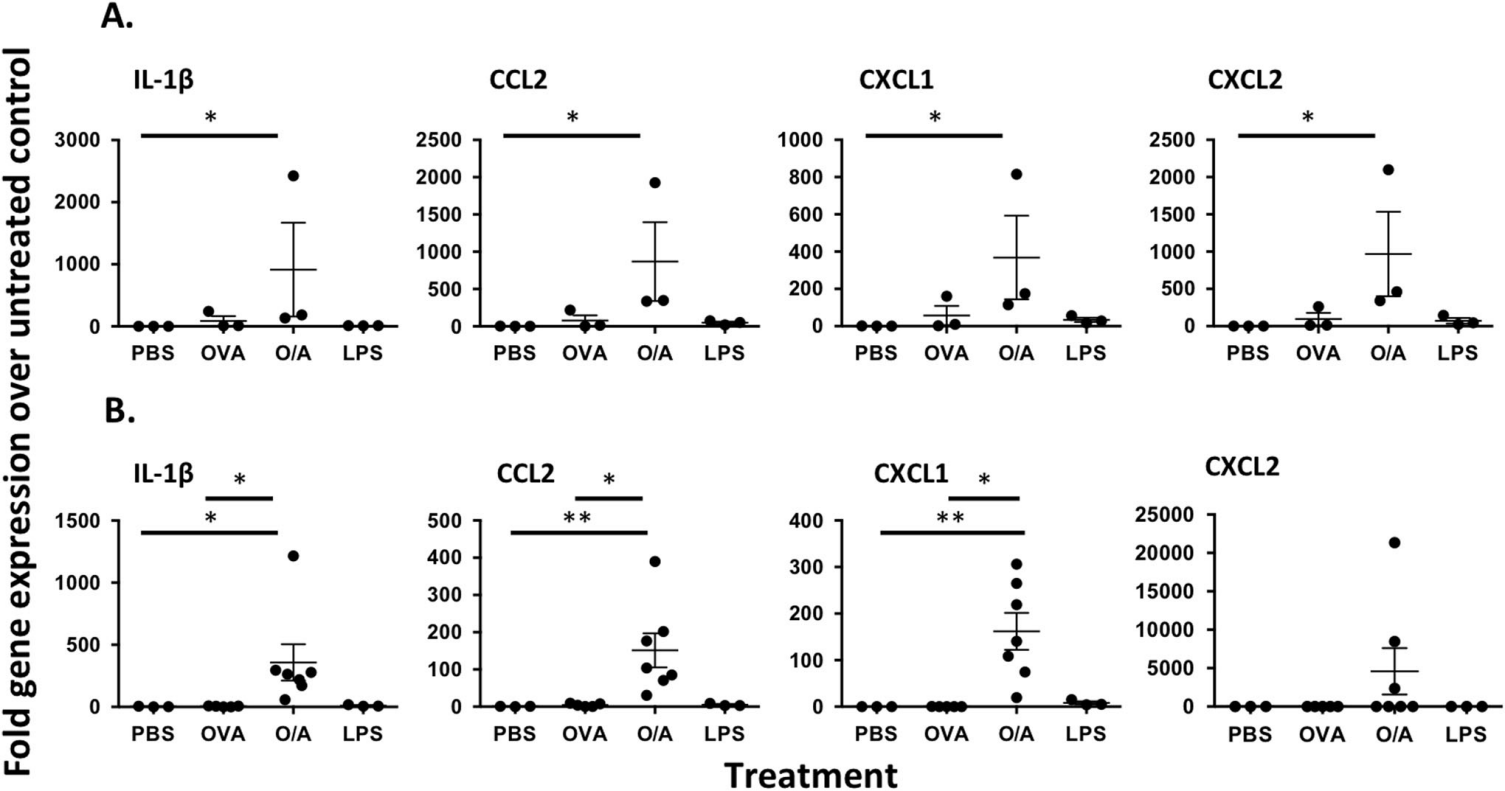
Inflammatory chemokine and cytokine expression at the site of immunisation and dLN 2 h after Alum injection. Representative examples of inflammatory chemokine and cytokine expression at **a** the site of alum injection (three a﻿nimals per group) and **b** draining cervical lymph nodes (three to seven animals per group). Data are representative of two independent experiments. *P<0.﻿05, **P < 0.01

Previous studies using ear pinna injection site ablation demonstrated that the injection site and any inflammatory reaction occurring therein was dispensable for adjuvant activity within 2 h post injection.^4^ Clearly, for any inflammatory response to play a role in adjuvant activity, it would have to occur within that narrow time frame. Analysis of Ly6G^+^CD11b^+^ neutrophils at the site of immunisation 2 h after ovalbumin (OVA)/Alum injection demonstrated an increased frequency (Fig. 1a) and number (Fig. 1b) of these cells at the site of immunisation compared with controls (UT; untreated control, PBS (phosphate-buffered saline); ears injected with PBS). The inflammatory site induced by Alum at this time was characterised by the presence of neutrophils, as there were no significant differences in the number of F4/80^+^ macrophages or CD11c^+^ DCs (Fig. 1b). In contrast with the site of immunisation, Alum did not induce neutrophil recruitment to the draining lymph node (dLN) compared with OVA-immunised controls 2 or 24 h following immunisation, whereas significant neutrophil recruitment could be found 2 h following lipopolysaccharide (LPS) injection (Fig. 1c, d). Analysis of inflammatory cytokine and chemokine transcription revealed significant increases in IL-1β, CCL2, CXCL1 and CXCL2 transcripts at the OVA/Alum injection site within 2 h (Fig. 2a). Furthermore, inflammatory responses were also evident within the dLN at this time point (Fig. 2b), indicating the rapid dissemination of the inflammatory response from the tissue to the draining LN. Together, these data demonstrate that Alum induces an intense and rapid inflammatory response at the injection site that quickly impacts on the dLN.

### Injection of Alum induces neutrophil recruitment and swarming at the site of immunisation

**Fig. 3:**
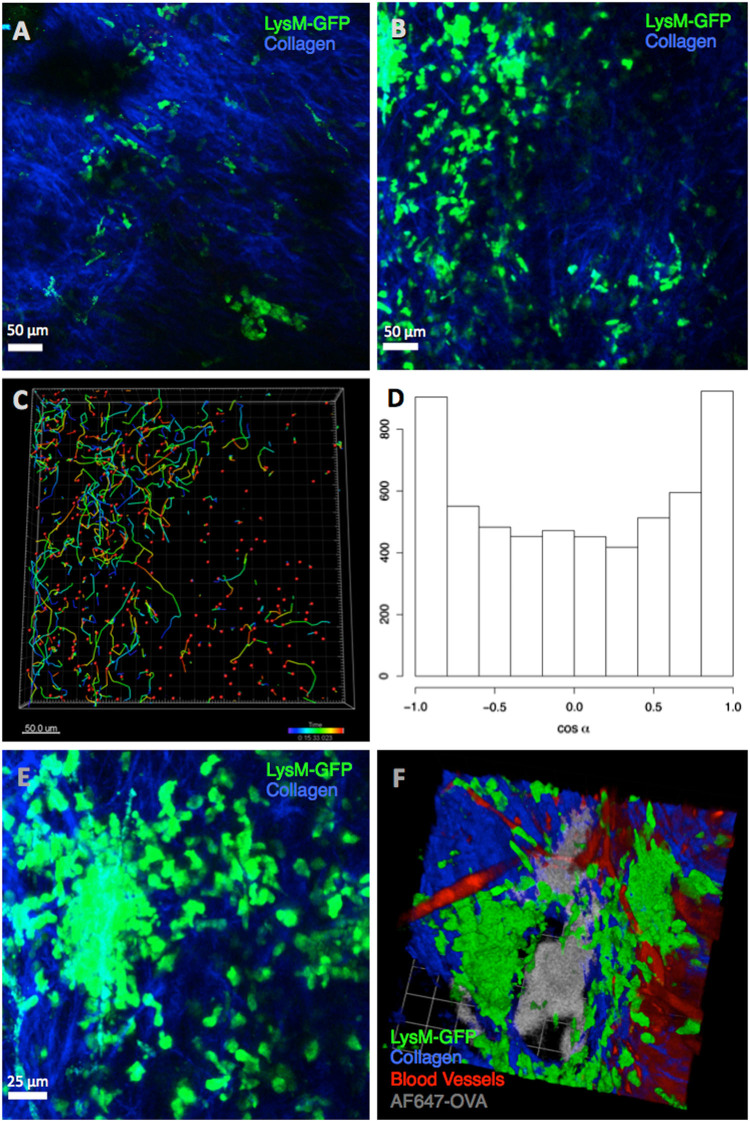
Alum induces neutrophil recruitment and swarming at the site of immunisation within 2 h. LysM-eGFP mice were injected with **a** OVA alone or **b** OVA/Alum 10 min prior to intravital MPLSM. While some neutrophil recruitment could be observed following injection of OVA, substantially more neutrophils were observed following OVA/Alum injection. **c** Tracking GFP+ neutrophils using a dragon plots demonstrated highly linear, directional movement in tissue and **d** this was quantified using analysis of turning angle. **e** This directional movement resulted in the formation of localised neutrophil ‘swarms’. **f** Immunisation with Alexa-647-labelled alum confirmed that the neutrophils were swarming around deposits of alum at the site of injection. Images shown are representative of three independent experiments

To investigate the cellular dynamics and behaviour of recruited neutrophils, we took advantage of the ability to visualise the Alum injection site in the ear pinna of LysM-eGFP reporter mice using multiphoton microscopy without the requirement for surgery.^12^ OVA alone or OVA/Alum was injected into the ear pinna of LysM-eGFP mice. While a small number of GFP+ neutrophils were observed at the OVA injection site (Fig. 3a; Supplementary Movie 1) treatment with OVA/Alum produced greatly enhanced neutrophil recruitment (Fig. 3b; Supplementary Movie 2) consistent with the flow cytometric analysis (Fig. 1a, b). The movement of cells in the OVA/Alum-treated mice also appeared different to that observed in control OVA-treated mice. The area around OVA/Alum injection sites contained neutrophils displaying highly directional, linear motility (Fig. 3c; Supplementary Movie 3) with a bimodal distribution of turning angle of 0° (directly towards) or 180° (directly away from) the Alum injection site (Fig. 3d). This migration resulted the formation of clusters of neutrophils (Fig. 3e; Supplementary Movie 4). The presence of Alum at the core of these clusters was confirmed using fluorescently labelled protein adsorbed to Alum (Fig. 3f; Supplementary Movie 5). The neutrophil identity of the GFP+ cells was confirmed by flow cytometry (FACS) analysis that demonstrated fourfold higher expression of GFP in neutrophils compared with monocytes and macrophages. This was reflected in the GFP dim, resident cells in untreated ears, presumably macrophages (Fig. 1b) that did not resemble the GFP bright, swarming neutrophils seen after Alum injection (Supplementary Figure 1). Overall, the imaging data demonstrate that Alum rapidly induces the recruitment of GFP+ neutrophils to the site of injection and that these neutrophils swarm around deposits of Alum found at the injection site.

### Alum induces neutrophil cell death and the release of extracellular DNA at the site of immunisation within two hours *in vivo*

**Fig. 4:**
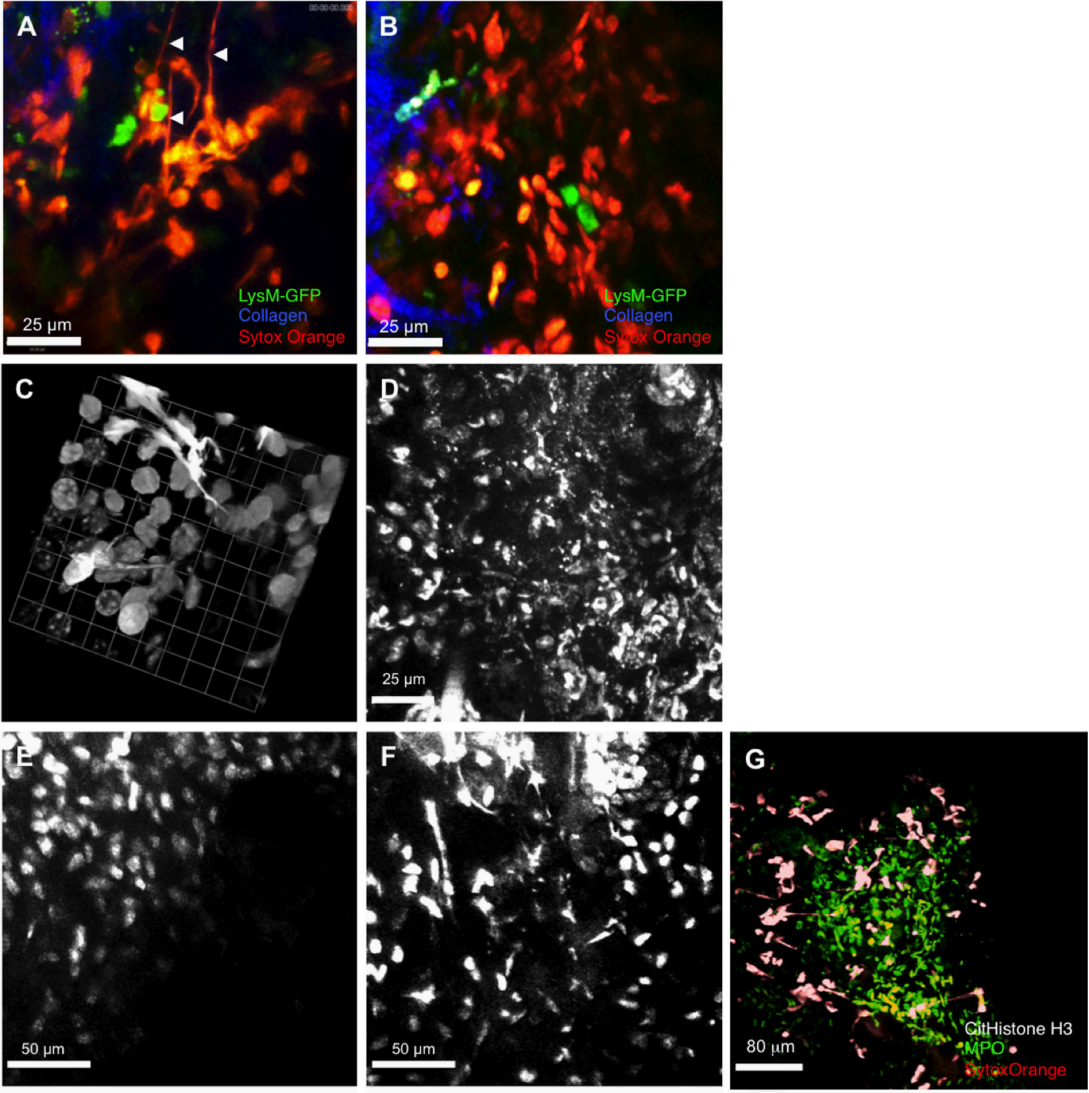
Alum induces cell death and the release of NETs at the site of immunisation. Cell nuclei became accessible to the cell-impermeant DNA dye, Sytox orange following **a** Alum/OVA or **b** OVA injection of LysM-eGFP mice. **a** Neutrophil (GFP+) nuclei only became Sytox orange-positive following Alum/OVA injection, and this was accompanied by the appearance of long strands of stained DNA (*arrowheads*). **c** High-resolution-rendered images using cell-permeant Hoechst 33342 nuclear dye at the injection site confirmed that these structures resembled NETs. **d** DNAse treatment at the site of alum immunisation degraded the extracellular DNA structures released from neutrophils, visualised with Hoechst 33342. Images in **a**, **b** are representative of *n* = 3, **c**, **d** of *n* = 5. (**e**) OVA/Alum does not induce the release of extracellular traps at the site immunisation in PAD4KO mice compared with **f** control littermates. NETs were visualised using Hoechst 33342 as above, and data shown are representative of five mice. **g** Histone H3 and DNA (Sytox Orange) co-localise within NETs, with a local release of myeloperoxidase evident at the injection site

Using the cell-impermeant DNA dye, Sytox Orange, we examined cell death and DNA release at the injection site in LysM-eGFP mice. Nuclear staining could be observed in GFP-positive neutrophils as well as GFP-negative cells, which, given the inflammatory profile established in Fig. 1, were presumably tissue-resident cells (Fig. 4a, b). Closer analysis of injection sites in Alum-treated mice (Fig. 4a) also revealed Sytox Orange-positive strands (arrowheads) that were not visible in OVA-treated animals (Fig. 4b). Higher-resolution, three-dimensional (3D)-rendered images of these extracellular DNA protrusions identified using Hoescht 33342 displayed the characteristic features of NETs (Fig. 4c). Treatment of OVA/Alum-immunised mice with DNAse resulted in loss of these structures, confirming the identity of these strands as extracellular DNA (Fig. 4d). Previous studies have demonstrated that deimination of histones H3 and H4 by peptidylarginine deiminase 4 (PAD4) is required for NET formation, and consequently neutrophils from PAD4ko mice fail to undergo netosis.^13,14^ PAD4ko mice therefore provided a means to confirm the identity of the Alum-induced strands as NETs, as well as an approach to reveal the functional role of netosis in Alum adjuvant activity. Intravital multiphoton laser scanning microscopy (MPLSM) analysis 2 h after OVA/Alum injection of PAD4ko mice failed to identify DNA strands (Fig. 4e) at the injection site, while these structures were abundant in PAD4 replete littermate mice (Fig. 4f), confirming their identity as NETs. Finally, immunohistochemistry of whole-mount ears following Alum injection revealed Histone H3 and DNA (Sytox Orange) co-localising within NETs, with a local release of myeloperoxidase evident at the injection site (Fig. 4g).

### Deletion of the PAD4 enzyme affects the polarisation of the adaptive immune response to alum adjuvant

**Fig. 5:**
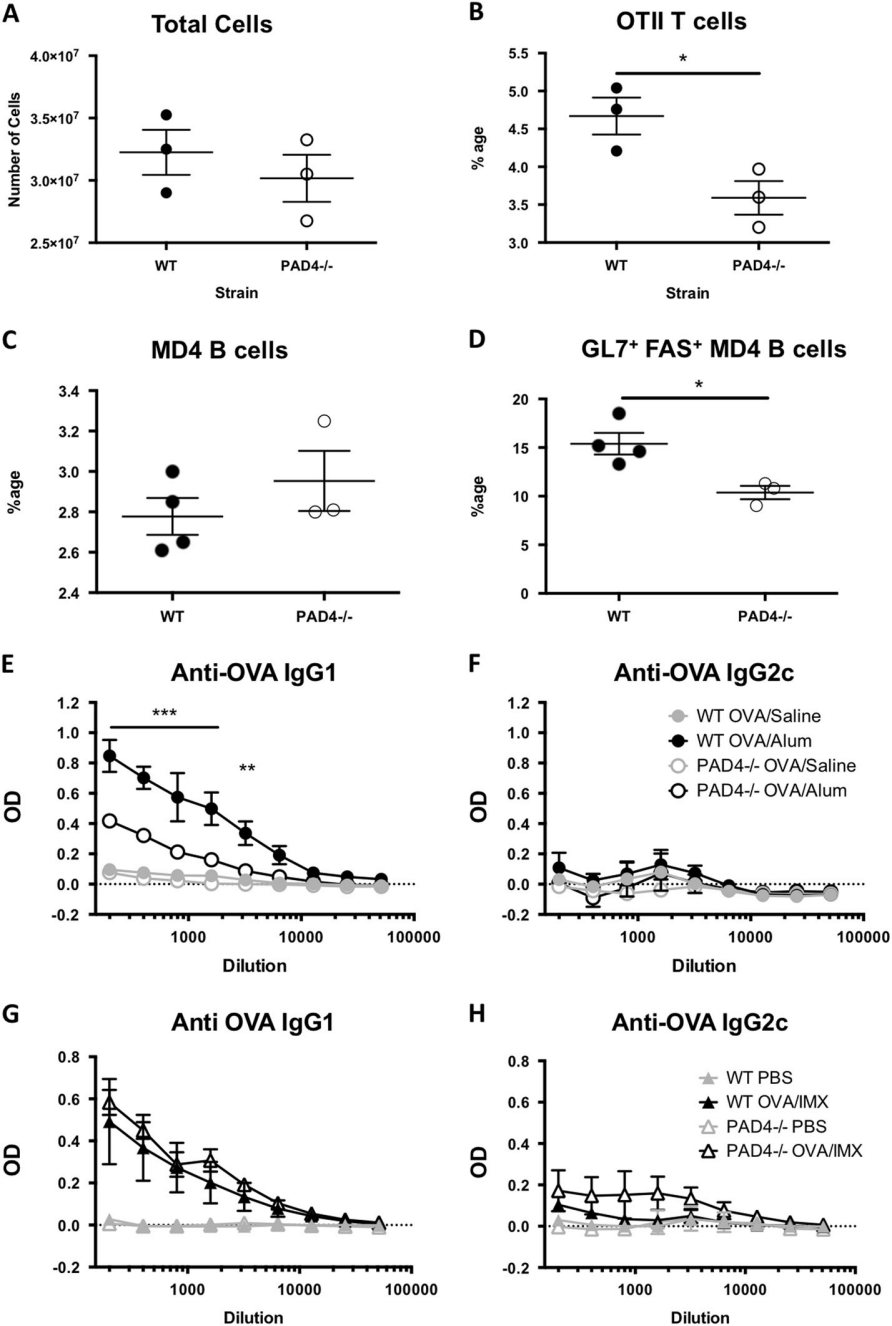
Deletion of PAD4 affects the magnitude of the adaptive immune response induced by alum adjuvant. Antigen-specific T (OTII) and B cells (MD4) were adoptively transfered to PAD4ko and WT mice. **a** Five days after immunisation, PAD4ko mice had similar numbers of cells in dLNs; **b** however, the proportion of antigen-specific OTII T cells was significantly lower in PAD4ko compared with WT mice (*P < 0.05). **c** Analysis of antigen-specific B cells on day 7 after immunisation revealed a similar proportion of the B-cell population in PAD4ko mice were IgMa+ HEL-specific; **d** however, the fewer GL7^+^ FAS^+^ germinal centre B cells were observed (*P < 0.05) compared with WT control mice. **e** PAD4KO mice immunised with OVA/Alum show reduced OVA-specific IgG_1_ titres compared with WT littermates 14 days after immunisation. **f** Levels of antigen-specific IgG_2c_ antibodies are undetectable in both strains of mouse. **g**, **h** Antigen-specific IgG_1_ and IgG_2a_ responses were similar in WT and PAD4KO mice 14 days after immunisation with OVA/ISCOMATRIX. Levels of antigen-specific IgG_2c_ antibodies are undetectable in both strains of mouse. Antibody analysis was performed on two to four mice per group, and data are representative of two to three experiments. *P<0.05, **P < 0.01, ***P < 0.001

Next we sought to determine whether the formation of NETs at the Alum injection site plays a role in driving the adjuvant activity of Alum by analysing antigen-specific T (OTII) and B cell (MD4) responses following adoptive transfer to PAD4ko and wild-type (WT) mice. At the peak of the antigen-specific T-cell response, 5 days after immunisation,^15^ PAD4ko mice had significantly (*P* < 0.05) lower OVA-specific, OTII T cells as a proportion of the T-cell population (Fig. 5b). Analysis of antigen-specific MD﻿4, B cells on day 7 after immunisation revealed that a similar proportion of the B-cell population in PAD4ko mice were IgMa+ HEL-specific (Fig. 5c), however, the ability of these cells to form GL7+ FAS+ germinal centre B cells was significantly lowered (*P* < 0.05) compared with WT control mice (Fig. 5d). Subsequently, we determined levels of IgG_1_, characteristic of Alum adjuvant activity and IgG_2c_, which is associated with a Th_1_ response. Immunisation of PAD4ko mice with OVA/Alum resulted in significantly decreased IgG_1_ antibody production compared to WT mice while IgG_2c_ production was unchanged (Fig. 5e, f). OVA-specific responses following immunisation with antigen prepared in an alternative adjuvant, ISCOMATRIX (IMX), were similar in both PAD4ko and control mice, indicating a specific loss of responsiveness to Alum adjuvant (Fig. 5g, h). Collectively, these studies demonstrate that PAD4-dependent netosis at the Alum injection site plays a significant role in the ability of Alum to act as an adjuvant that promotes B-cell differentiation and IgG_1_ antibody production.

## Discussion

Our studies demonstrate that Alum induces a rapid recruitment, swarming and PAD4-dependent netosis following injection and furthermore that this response is responsible for a significant portion of the adjuvant effect induced by Alum.

These findings are consistent with our previous studies demonstrating that key events in Alum adjuvant function occurred within the first 2 h following immunisation.^4^ Recruitment of neutrophils to the site of Alum injection was particularly rapid compared with LPS, observable within minutes of injection and reaching a peak within 2 h, consistent with previous reports in models of sterile inflammation.^16^ Following injection of antigen alone we observed a small increase in neutrophil recruitment in tissue by both FACS and intravital MPLSM; however, the presence of NETs was associated with a significant increase in neutrophil recruitment, suggesting NETs may act to amplify neutrophil recruitment by Alum, as previously observed in models of sterile tissue injury.^16^

Although first described as having an important role in innate immunity against bacteria,^17^ our data and those of previous studies^18,19^ support a role for NETs in shaping the adaptive immune response. NETs have been described in response to a number of infectious^20^ and sterile challenges^16,21,22^ *in vivo*; however, this is the first description of Alum inducing extracellular trap formation by neutrophils. Previous studies reported that Alum was able to induce fibrin extracellular traps at the site of immunisation,^23^ which were composed of fibrin, extracellular chromatin, citrullinated histones and were dependent on fibrinogen for formation. In contrast to our findings, Munks *et al*. suggested that these extracellular structures were independent of neutrophils and were not required for the adjuvant activity of Alum.^23^ This discrepancy with our current findings likely arises from the characterisation and location of these structures. Munks *et al.* focussed on the fibrin component, showing that neutrophil-depleted mice formed fibrin nodules without assessing the nucleic acid composition. Furthermore the intraperitoneal route of immunisation exposes a number of specialised resident cell populations that are not found in subcutaneous tissue,^24,25^ in particular the large peritoneal macrophages that disappear rapidly after inflammatory challenge.^24^ In the skin, neutrophils are the principal cell population recruited to the skin in response to Alum, and we now show for the first time these cells swarm towards Alum injection sites. PAD4 expression is broadly restricted to neutrophils and peritoneal macrophages within the murine immune system,^13,14,26^ and the absence of NETS observed in PAD4-deficient mice indicates that neutrophils are the most likely source of NETS forming in the skin in response to Alum.

A role for DNA released at the Alum injection site in adjuvant function has previously been demonstrated as treatment with DNAse following Alum immunisation reduced antigen-specific CD4 T-cell activation and IgG and IgE antibody responses.^10^ However, the cellular source and sensing of this DNA remained unclear. We now show that the very early inflammatory response to Alum drives neutrophil recruitment and generates free DNA via PAD4-dependent netosis. This response enhances antigen-specific T expansion and B-cell differentiation leading to an increased, class-switched antibody response. In the context of vaccination, these studies demonstrate that the later inflammation evident at the Alum injection site is superfluous for adjuvant activity and furthermore suggest that components released during the neutrophil netosis response may have utility as vaccine adjuvants.

## Methods

### Mice

Six to eight-week-old female BALB/c mice were purchased from Harlan (Bicester, UK). LysM-eGFP mice were a kind gift from Professor Sussan Nourshargh. These mice have the gene for eGFP knocked into the Lysozyme (Lys) M locus resulting in mice with fluorescent myelomonocytic cells, with neutrophils comprising the highest percentage of eGFP^hi^ cells.^12^ PAD4-floxed mice were a kind gift from Dr Kerri Mowen.^13^ To generate PAD4-KO mice and control littermate mice, PAD4-floxed mice were crossed with the ella cre deleter strain (B6.FVB-Tgn(Ella-Cre)C5379Lmgd (Charles River, UK). Male and female PAD4-KO mice and control littermate mice were used at 7–16 weeks of age. OT-II TCR Tg mice^27^ and MD4 BcR Tg mice^28^ on C57BL/6 backgrounds were used as a source of OVA-specific T cells and HEL-specific B cells, respectively. All mice were either injected subcutaneously (s/c) in the ear pinna with 10 μl or in the scruff with 100 μl of antigen/adjuvant suspensions. Mice were immunised with either 100 μg of chromatographically purified chicken OVA (Worthington Biochemical, Lakewood, NJ, USA), or OVA-HEL conjugate prepared using glutaraldehyde to couple the chicken OVA and HEL (Sigma, Dorset, UK) as published.^15^ Antigens were prepared in a 1% Alum suspension (a gift from Dr Erik Lindblad﻿, Brenntag Biosector, Denmark) or in 1IU ISCOMATRIX (a kind gift from CSL Limited, Australia) or 1 μg LPS (*E. coli* 0111:B4; Sigma Aldrich, UK). To identify the site of injection, mice were also injected Alexa-647-labelled OVA prepared using an Alexa-647 protein labelling kit (Thermo Fisher Scientific) according to the manufacturer’s instructions. All buffers and media were purchased endotoxin-free and absence of appreciable endotoxin contamination of proteins was confirmed by absence of bone marrow-derived dendritic cell activation at a dose of 500 µg. Animals were allocated to experimental groups by a blinded observer, and were maintained under standard animal house conditions at the University of Glasgow and procedures were performed according to UK Home Office regulations.

### Taqman low-density arrays

Ear tissue and superficial cervical dLNs were excised and stored in RNA*later* (Qiagen). RNA was extracted using the RNeasy mini kit (Qiagen) according to the manufacturer’s instructions and was homogenised using a gentleMACs Dissociator (Miltenyi Biotech, UK). RNA was converted to cDNA using a reverse transcription kit from Primer Design (Primer Design Ltd, Southampton, UK). One μg of cDNA was then loaded onto customised Taqman low-density array (TLDA) plates (Applied Biosystems, Life Technologies, Paisley, UK) and probed for the presence of cytokine and chemokine genes. Samples were subsequently analysed on an Applied Biosystems 7900HT Fast Real-Time PCR System, using the SDS2.4 software. Data were analysed on the RQ Manager 1.2.1 software. Data are shown as fold change over the appropriate untreated control tissue. For analysis of the injection site (ear) *n* = 3 for each treatment group; for analysis of the dLNs *n* = 5 for the OVA-treated group, *n* = 7 for the OVA/ALUM-treated group and *n* = 3 for the untreated, the PBS and the LPS-treated groups. Where a target transcript was undetectable an arbitrary value of 0.001 was assigned. The data shown are representative of two independent experiments.

### Flow Cytometry

Cell suspensions were prepared from ear tissue by digestion in 2 mg/ml Collagenase IV and 2 mg/ml Hyaluronidase (both from Sigma Aldrich) and DNaseI (100 U/ml; Invitrogen) at 37 °C for 30 min, followed by homogenisation using a gentleMACs Dissociator (Miltenyi Biotech). The draining superficial cervical lymph node was gently teased apart using 26G needles and digested in 2.68 mg/ml collagenase D (Roche) for 25 min at 37 °C. The enzymatic reaction was stopped with a final concentration of 10 mM EDTA. A single cell suspension was then obtained by passing the lymph node suspension through a 70 μm cell strainer. Single cell suspensions were then stained with combinations of the following antibodies CD11c–PerCP-Cy5.5 (N418; Cat. No. 45-0114; eBioscience, Hartfield, UK), CD11b–PE-Cy7 (M1/70; Cat. No. 25-0112; eBioscience), F4/80–APC (BM8; Cat. No. 123116; BioLegend, San Diego, USA) CD45-eFluor®450 (30-F11; Cat. No. 48-0451; eBioscience), Ly6-G–PE (IA8; Cat. No. 127608; BioLegend), MHCII–FITC (2G9; Cat. No. 553623; BD Biosciences, UK), anti-B220-eFluor®450 (RA3-6B2; Cat. No. 48-0452; eBioscience), anti-GL-7-FITC (GL7; Cat. No. 553666 BD Biosciences), anti-FAS-PE-Cy7 (Jo2; Cat. No. 557653; BD Biosciences), anti-CD45.1-APC (A20; Cat. No. 558701; BD Biosciences), anti-CD4-PE-Cy5 (GK1.5; Cat. No. 100409, BioLegend), anti-Vb5.1/5.2-PE (MR9-4; Cat. No. 553190; BD Biosciences) and biotinylated anti-IgMa (DS-1; Cat. No.553515; BD Biosciences). Streptavidin-APC-eFluor®780 (Cat. No. 47-4317; eBiosciences) was used to detect the biotinylated antibody. The viability of the cells was confirmed using a fixable viability stain conjugated to eFluor®450 (eBioscience). All antibodies and isotype controls were added to samples at a dilution of 1:200. Samples were read using a MACSQuant flow cytometer (Miltenyi Biotech, UK) and analysed using Flowjo software (Version 9.4 or 10.0.8; Treestar). For analysis of innate cell recruitment to the injection site group sizes of six mice were used. The data shown are representative of three independent experiments. For analysis of innate cell recruitment to the dLN a group size of four was used for OVA treatment; five for OVA/ALUM treatment and three for LPS treatment. Using data from previous studies^29^ we estimated three mice would provide 80% power to detect significant differences (*a* < 0.05) in numbers of neutrophils recruited to tissue in response to adjuvant vs. PBS control. For the analysis of T-cell transfer and B-cell transfer into PAD4KO and WT mice on day 5 the group size was *n* = 3 and on day 7 the group size was *n* = 4 for WT mice and *n* = 3 for PAD4KO mice. The data shown are representative of two independent experiments. Using previous data,^30^ we estimated three mice per time point will provide 80% power to detect significant differences (*a* < 0.05) in numbers of Plasma B cells induced by antigen and adjuvant vs. antigen alone.

### Enzyme linked immunosorbent assay

OVA-specific IgG_1_ and IgG_2a_/IgG_2c_ titres were determined in serum samples collected 14 days after immunisation as previously described.^2^ For the analysis of antibody responses to OVA/ALUM the group sizes were *n* = 3 while for the analysis of antibody responses to OVA/IMX the group sizes were *n* = 4 with *n* = 2 for PBS-treated controls. The data shown are representative of three independent experiments.

### *In vivo* multiphoton imaging

Multiphoton imaging was performed with a Zeiss LSM7 MP system equipped with both a 10×/0.3 numerical aperture (NA) air and a 20×/1.0 NA water immersion objective lens (Zeiss) and a tunable titanium/sapphire solid state two-photon excitation source (Chamelon Ultra II; Coherent Laser Group). For *in vivo* imaging, animals were anaesthetised with fentanyl/fluanisone (Hypnorm: Janssen Animal Health) and midazolam injectable anaesthesia (Hypnovel: Roche; 10 ml/kg of an aqueous mix of fentanyl/fluanisone/ midazolam at 1:1:2 by volume) given intraperitoneally. Core body temperature was continuously monitored and maintained by a thermostatically controlled heat mat. The ear of interest was mounted on a purpose-built, heated stand with the use of veterinary-grade glue (Vetbond; 3M), enabling maintenance of tissue at 37 °C throughout the experiment.^31^ Intravenous injection of non-targeted quantum dots (Qtracker 655 and 705; Invitrogen) allowed highlighting of blood vessels. Cells were visualised in the ear pinna using the cell-permeable DNA dye Hoechst 33342 (Sigma Aldrich, UK) and extracellular DNA visualised with SYTOX orange (Molecular Probes, Invitrogen, Paisley, UK). The dyes were administered subcutaneously along with the antigen/adjuvant preparations. Mice were also treated subcutaneously in the ear pinna with 5 mg of DNase along with antigen/adjuvant preparations to block NET formation at the injection site.^17^ Videos were acquired for 15–30 min at an X–Y pixel resolution 512 × 512 with 2.5-μm increments in Z. 3D tracking was performed with both Imaris 7 (Bitplane) and Volocity 5.5 (Perkin Elmer) after correction for tissue drift.

### Whole-mount staining

Ears were carefully split into dorsal and ventral halves and fixed in 2% paraformaldehyde prepared in PBS (PFA/PBS) (eBiosciences) for 30 min on ice. Fixed ears where then permeabilised in 0.2% Triton X-100 (Sigma)/PBS on ice for 15 min, and blocked overnight at 2–8 °C with 10 µg/ml anti-CD16/CD32 (2.4G2; Cat. No. 553142; BD Biosciences), 5% Normal mouse serum, 0.2% Triton X-100 in PBS. FITC conjugated anti-MPO (2D4; Cat. No. ab90812; Abcam, UK) and purified rabbit anti-citrulinated Histone H3 (Polyclonal; Cat. No. ab5103; Abcam, UK) were added to give a final concentration of each at 2 µg/ml and incubated at 2–8 °C overnight. Tissues were washed in three changes of 0.2% triton X-100/PBS at 2–8 °C for 1 h. The Alexafluor 647-conjugated secondary antibody (Goat anti-Rabbit IgG; Cat. No. ab150083; Abcam) was added at 2 µg/ml in 2% NMS/0.2% Triton X-100/PBS overnight at 2–8 °C. Tissues were washed in three changes of 0.2% Triton X-100/PBS at 2–8 °C for 1 h. Sytox Orange was added at a 1/10,000 dilution in 0.2% Triton X-100/PBS and incubated at 2–8 °C for 1 h. Tissues were then fixed in 1% PFA/PBS and imaged on the multiphoton microscope.

### Statistics

For flow cytometry and enzyme linked immunosorbent assay data, intergroup significance was determined by either a one-way ANOVA or a two-way ANOVA with a Tukey post test. For TLDA data intergroup significance was determined using the non-parametric Kruskal–Wallis test with a Dunns post test. Statistical analysis was performed using GraphPad Prism 6 (GraphPad Software Inc., La Jolla, USA). A value of *P* ≤ 0.05 was considered significant.

## Electronic supplementary material

Supplementary Figure 1

Supplementary Movie 1

Supplementary Movie 2

Supplementary Movie 3

Supplementary Movie 4

Supplementary Movie 5
