# Supplementary figures and images for: Neutrophil swarming and extracellular trap formation play a significant role in Alum adjuvant activity
Source: NPJ Vaccines. 2017 Jan 23;2:1. doi: 10.1038/s41541-016-0001-5 (PMC5604741; doi:10.1038/s41541-016-0001-5)

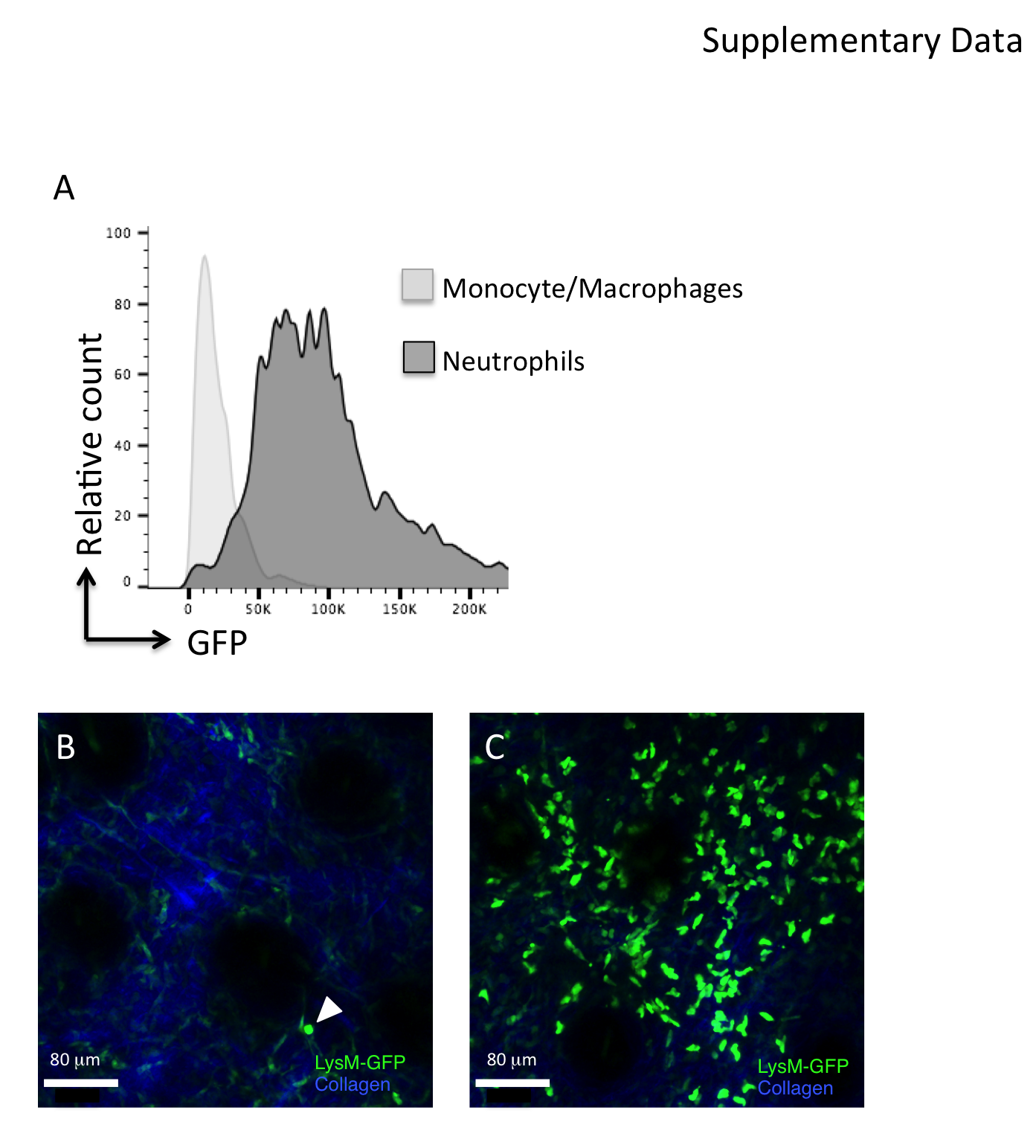

Supplement: Supplementary file 1 — Supplementary Figure 1 [file 41541_2016_1_MOESM1_ESM.tif]
